# Restrictive ID Policies: Implications for Health Equity

**DOI:** 10.1007/s10903-017-0579-3

**Published:** 2017-04-08

**Authors:** Alana M. W. LeBrón, William D. Lopez, Keta Cowan, Nicole L. Novak, Olivia Temrowski, Maria Ibarra-Frayre, Jorge Delva

**Affiliations:** 1 Program in Public Health, University of California, Irvine, 653 E. Peltason Drive, Anteater Instruction and Research Building, Room 2026, Irvine, CA 92697-3957 USA; 2 Department of Chicano/Latino Studies, University of California, Irvine, Irvine, CA USA; 3 School of Social Work & National Center for Institutional Diversity, University of Michigan, Ann Arbor, MI USA; 4 Synod Community Services, Ypsilanti, MI USA; 5 Population Studies Center, Institute for Social Research, University of Michigan, Ann Arbor, MI USA; 6 School of Social Work & Michigan Institute for Clinical and Health Research, University of Michigan, Ann Arbor, MI USA

“I got close to see what they were giving out, and it was water. And the first thing they asked me for was my license.”—Resident of Flint [1]

The need for an ID to access publicly-distributed bottled water following the lead water contamination crisis in Flint, Michigan, serves as just one example of the increasingly critical link between photo identification (ID) issued by US governmental entities (henceforth, *government-issued IDs*) and access to health-promoting resources. As advocates and researchers for a local government-issued ID in Washtenaw County, Michigan, the first county ID issued in the Midwest, we argue that post-9/11 policies and practices that expand the domains in which a government-issued ID is required and increase the barriers to obtaining these IDs have profound implications for health and health equity. Government-issued IDs are increasingly required to access a number of health-related resources. These resources include goods and services (e.g., purchasing with a debit/credit card or check) [2]; financial (e.g., getting a bank account, cashing a check; applying for a loan); identification or governmental records (e.g., obtaining a birth certificate or marriage license); health care and pharmaceutical (e.g., proving identification to a health care provider, applying for health insurance or sliding scale health care fees, purchasing prescribed medications) [3]; community (e.g., applying for a library card, participating in safety net programs such as food banks) [4]; caregiving (e.g., picking up children from school); housing (e.g., viewing an apartment, completing a lease); political enfranchisement (e.g., registering to vote, participating in an election) [5, 6]; and the ability to remain in the US with family and community (e.g., identifying self to police or immigration officials) [7].

## Restrictive ID Policies Trickle Down to Health Inequities

A number of communities encounter barriers to acquiring government-issued IDs. A decade ago, an estimated 21 million voting-age US citizens (11%) lacked unexpired government-issued ID, of whom non-Latino Black adults (25%), Latino adults (16%), adults with incomes <$35,000 annually (15%), and persons age 65 or older (18%) were disproportionately impacted [8]. Other communities are also affected. Undocumented immigrants are barred from accessing government-issued IDs in most states [9]. Additional communities marginalized by restrictive ID policies include those navigating residential instability, homelessness, or catastrophic events (e.g., fire, environmental disaster, domestic violence) for whom proof of residence and identity, generally a requirement for an ID, may be impossible [10]. Health status also structures access to government-issued IDs: individuals with chronic mental illness may experience residential instability and subsequent difficulty in proving identity and residency. As government-issued IDs include a person’s legal name and biological sex assigned at birth, transgender individuals may encounter difficulties acquiring IDs when their gender presentations do not match their sex assigned at birth [11]. Older Black adults born during the Jim Crow era may have never received a birth certificate that verifies their identities or US citizenship. Additionally, formerly incarcerated individuals are often released from prison without documents that would assist them in obtaining a government-issued ID, exacerbating the challenges of re-integrating into their communities [12].

## Restrictive ID Policies Shape Access to Health-Promoting Resources

We detail three mechanisms through which post-9/11 policies have expanded the instances in which government-issued ID requirements have restricted access to health-promoting resources. These policies augment an intricate web of institutional discrimination to shape the social conditions and health of multiple marginalized communities.

First, the 2001 PATRIOT Act [13] codified into law several provisions that require verification of identity to access certain economic and material resources. For example, the PATRIOT Act’s Customer Identification Program mandates that financial institutions implement institutional policies to verify the identities of their customers. In response, many banks and creditors (e.g., utilities companies) have enacted policies that require a government-issued ID (which has typically been interpreted as a US passport, state ID, or state driver’s license) when opening a bank account, cashing a check, or acquiring utilities. However, the PATRIOT Act allows each institution to exercise discretion in determining the types of ID required. Thus, the effects of this federal policy are compounded by many financial institutions’ exclusionary interpretation of the identity verification measures mandated by the PATRIOT Act.

Second, federal policies requiring identity verification spill over to impede access to health care and other health-promoting resources such as nutritional assistance and clean water. Indeed, several policies (e.g., PATRIOT Act’s Customer Identification Program) and concerns about medical identity theft [14] have prompted health care providers and administrators to request a government-issued ID when providing and billing for services. Furthermore, the Deficit Reduction Act of 2005 [15] mandated proof of identity and citizenship in accessing Medicaid, contributing to a decline in Medicaid enrollment [16]. The Deficit Reduction Act of 2005 built on and expanded the restrictions created by the 1996 Personal Responsibility, Work Opportunity and Reconciliation Act (PRWORA) [17]. PRWORA implemented a 5-year bar in eligibility for food stamps, Medicaid, and Supplemental Security Income for recent immigrants with authorized US presence [17]. Consequently, PRWORA induced a “chilling effect,” or a reduction in utilization of social and health care services to which individuals and families were entitled, which spilled over to prevent US-born Latino children of immigrants from accessing social services and resources for which they were eligible [18]. By prohibiting access to these resources for immigrants who arrived after the law was passed and enforcing a wait period for recent immigrants, PRWORA reinforced and exacerbated citizenship and nativity-based privileges and racial inequalities [18–20]. Like PRWORA, by restricting access to health care to persons who can provide the requisite IDs, the Deficit Reduction Act of 2005 expands inequities in rights and access to resources between citizens and non-citizens and children of immigrants, and between those who have a current government-issued ID and those who do not.

The reach of these policies extends to the practices of organizations charged with promoting the public’s welfare who, through de facto practices, often require a government-issued ID to access these resources (e.g., library card, food assistance, holiday gifts for children). Consequently, emergency response measures, such as water distribution centers’ requirement of a government-issued ID to access bottled water in the early days of the Flint water crisis, deterred and excluded communities marginalized by restrictive ID policies from obtaining safe water. These practices by governmental institutions and non-profit organizations may be motivated by their need to document the number of unique individuals served or to ensure that they are delivering services to residents within their geographic region. While these entities may not have fully considered the consequences of institutional policies or practices that require government-issued IDs, such practices may augment the barriers to accessing health-related resources for communities who are disenfranchised by restricted access to government-issued IDs.

Third, federal policies restrict access to government-issued IDs in the first place. The 2005 REAL ID Act [21] established a national standard for identification credentials, mandating proof of authorized US presence in order for state-issued IDs to be used for federal identification purposes. As state-issued IDs (i.e. state IDs, state driver’s licenses) are the primary forms of identification used in many daily activities, the REAL ID Act burdens states with issuing IDs that meet these criteria [21]. Consequently, 40 states have implemented policies to deny state IDs and state driver’s licenses to persons who cannot prove or do not hold documented status or who cannot prove their state residency [9]. Collectively, these policies render the verification of an affirmed identity as a government-controlled resource.

Policies that restrict access to government-issued IDs (e.g., REAL ID Act, state interpretations of the REAL ID Act) disproportionately affect immigrant communities [22, 23]. As government-issued IDs have emerged as proof of authorized US presence, for immigrants who lack government-issued IDs, encounters with law enforcement agencies or other governmental entities may escalate to immigration-related detention or deportation [7, 22, 24].

Restrictive ID policies are one layer of the complex web of twenty-first century restrictive immigration policies that immigrants, particularly racial minority immigrants, navigate. The early twenty-first century has been characterized by several federal-, state-, and local-level restrictive immigration policies and practices, such as collaboration between federal immigration enforcement agencies and local law enforcement agencies, immigration raids, and state-level policies such as Arizona’s SB 1070 that criminalize the failure to present immigration documents [24–27]. A growing evidence base links restrictive immigration policies with racialized stressors [7, 28–30], restricted access to health-promoting resources [7, 20, 29–33], and adverse health outcomes [28, 34] for immigrant and US-born Latinos, the racial group that has been disproportionately burdened by restrictive immigration policies [26, 35].

## A Call for Further Research on the Health Equity Implications of ID Policies

Heightened barriers in accessing government-issued IDs and expanded domains in which a government-issued ID is required shape access to health-promoting resources and contribute to health inequities. As advocates working to reinstate access to government-issued IDs for all, we identify two critical and urgent areas of public health research. First, research, in partnership with communities affected by restrictive ID policies, is urgently needed to elucidate the relationship between ID policies and health equity. For example, research is warranted regarding the health equity implications of restrictive state-issued ID policies as well as inclusive state-issued ID policies in the 10 states that extend ID access to undocumented immigrants and others marginalized by restrictive ID policies [9].

Second, researchers could build upon the growing social movement to implement local government-issued IDs (henceforth, *local IDs*) [36–38] as a community-driven intervention to intervene upon the links between restrictive ID policies and health equity. This movement builds on examples of promising policies issued by local governments in New Haven, CT; Oakland and San Francisco, CA; Newark, NJ; and Washington, D.C [36]. More recently, in 2015 New York City, NY; Washtenaw County, MI; and Johnson County, IA implemented local ID policies, and in 2016 Detroit, MI and Milwaukee, WI began issuing municipal IDs. These local ID policies reflect initiatives to ensure that all residents have access to IDs that verify their identities and which can be utilized to access resources [36].

Evaluations of local ID policies may inform the evidence base of promising community-driven strategies to interrupt or mitigate the health and health equity implications of restrictive federal and state ID policies. Additionally, evaluations of current local ID policies may inform local ID policy considerations by other communities. This team of advocates and researchers is engaged in a mixed-methods evaluation of the Washtenaw County ID, which was passed in November 2014 and became available to all residents on June 1, 2015. To date, 1274 Washtenaw County residents have obtained their Washtenaw IDs. The Washtenaw ID process and outcome evaluation considers whether the Washtenaw ID is reaching communities most marginalized by restrictive ID policies as well as residents who hold current government-issued IDs. Additionally, through this evaluation we are examining whether the Washtenaw ID functions to provide access to social, economic, and psychosocial resources that are increasingly continent upon having government-issued IDs, and variations in these experiences. Specifically, the Washtenaw ID evaluation tests the hypothesis that local IDs may enhance access to social, economic, health care, and caregiving resources; improve access to goods and services; mitigate interactions with law enforcement agents by enabling residents to present forms of ID recognized by these agencies; and buffer the psychosocial toll of lacking identification-based recognition under the context of restrictive state ID policies. To test these hypotheses, we engage several research methods including baseline and follow-up surveys with Washtenaw ID holders who obtained their Washtenaw ID within the first 6 weeks of the Washtenaw ID’s availability; baseline interviews with Washtenaw ID holders; and a mystery shopper study evaluating racial variation in carding experiences and acceptance of the Washtenaw ID when purchasing goods.

If local ID policies serve ID holders as outlined above, they may also have health implications for ID holders’ kin and social networks. For example, individuals may use their local IDs to acquire resources (e.g., medication, emergency resources such as lead-free water, bank account) that enhance their and their network members’ health, social, and economic status. Additionally, ID holders may use their local ID to acquire resources for their network members (e.g., child’s passport or birth certificate). Thus, as several marginalized populations experience contested access to US government-issued IDs, local IDs have the potential to improve health equity for ID holders and their network members. Evaluations of this growing social movement for local IDs may shed light on the potential of local community policies to disrupt the social and health equity implications of restrictive federal and state ID policies.

## Restrictive ID Policies: A Public Health Call to Action

Finally, we must ensure that institutions provisioned with promoting the public’s health do not require government-issued IDs to access their services and resources. Accordingly, we must educate institutions about the role of government-issued ID policies in restricting access to resources provisioned by these organizations. Public health departments, organizations, and their partners (e.g., food banks, other service-providing organizations) need to identify strategies to inclusively serve affected communities while not invoking or exacerbating restrictive ID policies. Welcoming other forms of ID, such as non-profit IDs, school IDs, and non-US issued IDs, is an initial step in this direction.

In Washtenaw County, the Washtenaw ID Task Force has engaged public health and other governmental stakeholders and representatives of communities affected by restrictive ID policies in the process of developing and implementing the Washtenaw ID. Accordingly, the Washtenaw County Health Plan, which provides health insurance to low-income residents of Washtenaw County, has accepted the Washtenaw ID in residents’ applications for health insurance coverage through the County and has promoted the Washtenaw ID amongst residents. Recognizing the linkages between IDs, health care access, and access to needed medications, two community health centers and some local pharmacies in Washtenaw County have implemented a policy to accept the Washtenaw ID as proof of identity when obtaining health care, prescribed medications, or regulated over-the-counter medications, respectively. Additionally, the Washtenaw County Clerk’s office accepts the Washtenaw ID from residents who are obtaining a birth certificate, applying for a marriage license, and registering to vote. Notably, all law enforcement agencies within Washtenaw County have agreed to honor the Washtenaw ID as proof of identity, an important commitment to disrupting the linkages between interactions with law enforcement and detainment. Further, some local banks have agreed to accept the Washtenaw ID as the primary form of ID for individuals wishing to open a bank account. The Washtenaw ID Task Force and members of the Washtenaw ID evaluation team have worked to educate social service organizations such as food banks about the restrictive role of policies that require a government-issued ID and to encourage them to accept and promote the Washtenaw ID. These practices and partnerships serve to improve access to health-promoting resources for all residents, and in particular those most affected by restrictive ID policies.

In better understanding the linkages between restrictive ID policies and health, we hope that public health organizations and leaders will review their policies and practices and inform partners about the exclusionary consequences of policies that restrict access to health-promoting resources based on one’s ability to present a government-issued ID. In the weeks following initial efforts to publicly distribute bottled water in Flint, authorities learned about the role of policies mandating a state ID or driver’s license in restricting residents’ access to clean, potable water. The State of Michigan later released an announcement indicating that government-issued IDs were not required for Flint residents to access bottled water distributed by the government [39, 40]. However, initial practices of restricting access to clean water based on ID status served to augment mistrust towards the government for communities doubly marginalized by the water crisis and the government’s and partner organizations’ initial distribution of water to only those residents with government-issued ID. In learning from this example, we hope that governmental and social institutions charged with provisioning health-promoting goods and resources to residents will interrupt the linkages between government-issued IDs and access to health-related goods. This may involve removing requirements of a government-issued ID or allowing residents to use other strategies to identify themselves (e.g., local ID, consular ID, student ID, letter mailed to their home, verification of identity by another individual).

It is imperative that we strengthen our resolve to advocate for policies that will promote social and health equity and identify strategies to disrupt the linkages between restrictive ID policies and health.
